# GPR43 deficiency protects against podocyte insulin resistance in diabetic nephropathy through the restoration of AMPKα activity

**DOI:** 10.7150/thno.56598

**Published:** 2021-03-04

**Authors:** Jian Lu, Pei Pei Chen, Jia Xiu Zhang, Xue Qi Li, Gui Hua Wang, Ben Yin Yuan, Si Jia Huang, Xiao Qi Liu, Ting Ting Jiang, Meng Ying Wang, Wen Tao Liu, Xiong Zhong Ruan, Bi Cheng Liu, Kun Ling Ma

**Affiliations:** 1 Institute of Nephrology, Zhongda Hospital, School of Medicine, Southeast University, Nanjing, 210009, China.; 2 John Moorhead Research Laboratory, Department of Renal Medicine, University College London (UCL) Medical School, Royal Free Campus, London, NW3 2PF, UK.

**Keywords:** diabetic nephropathy, podocyte insulin resistance, GPR43, AMPKα activity, gut microbiota dysbiosis

## Abstract

**Rationale:** Albuminuria is an early clinical feature in the progression of diabetic nephropathy (DN). Podocyte insulin resistance is a main cause of podocyte injury, playing crucial roles by contributing to albuminuria in early DN. G protein-coupled receptor 43 (GPR43) is a metabolite sensor modulating the cell signalling pathways to maintain metabolic homeostasis. However, the roles of GPR43 in podocyte insulin resistance and its potential mechanisms in the development of DN are unclear.

**Methods:** The experiments were conducted by using kidney tissues from biopsied DN patients, streptozotocin (STZ) induced diabetic mice with or without global GPR43 gene knockout, diabetic rats treated with broad-spectrum oral antibiotics or fecal microbiota transplantation, and cell culture model of podocytes. Renal pathological injuries were evaluated by periodic acid-schiff staining and transmission electron microscopy. The expression of GPR43 with other podocyte insulin resistance related molecules was checked by immunofluorescent staining, real-time PCR, and Western blotting. Serum acetate level was examined by gas chromatographic analysis. The distribution of gut microbiota was measured by 16S ribosomal DNA sequencing with faeces.

**Results:** Our results demonstrated that GPR43 expression was increased in kidney samples of DN patients, diabetic animal models, and high glucose-stimulated podocytes. Interestingly, deletion of GPR43 alleviated albuminuria and renal injury in diabetic mice. Pharmacological inhibition and knockdown of GPR43 expression in podocytes increased insulin-induced Akt phosphorylation through the restoration of adenosine 5'-monophosphate-activated protein kinase α (AMPKα) activity. This effect was associated with the suppression of AMPKα activity through post-transcriptional phosphorylation via the protein kinase C-phospholipase C (PKC-PLC) pathway. Antibiotic treatment-mediated gut microbiota depletion, and faecal microbiota transplantation from the healthy donor controls substantially improved podocyte insulin sensitivity and attenuated glomerular injury in diabetic rats accompanied by the downregulation of the GPR43 expression and a decrease in the level of serum acetate.

**Conclusion:** These findings suggested that dysbiosis of gut microbiota-modulated GPR43 activation contributed to albuminuria in DN, which could be mediated by podocyte insulin resistance through the inhibition of AMPKα activity.

## Introduction

Diabetic nephropathy (DN) represents the leading cause of end-stage renal disease (ESRD) in the world 1. Albuminuria is one of the dominant clinical manifestations during the development of DN. The presence of albuminuria means that the glomerular filtration barrier (GFB) has been damaged in early DN. Podocytes are the main cellular component of GFB. Podocyte injury, characterized by fusion, loss of the foot processes, and apoptosis, is a key cytological event leading to albuminuria during the progression of DN 2.

Insulin resistance is one of the main causes of podocyte injury in DN 3. Physiologically, in response to insulin stimulation, podocytes activate insulin receptor and the downstream phosphoinositide 3-kinase (PI3K)/Akt cascades to maintain podocyte viability and GFB integrity 4, 5. On the contrary, insulin resistance increases the risk of renal dysfunction in DN. Musso *et al.* reported that the mice with podocyte-specific insulin receptor knockout develop albuminuria, effacement of the podocyte foot process and glomerulosclerosis 6. Thus, podocyte-specific insulin signalling is crucial for glomerular function.

Recently, G protein-coupled receptor 43 (GPR43) has been investigated due to its involvement in energy modulation and insulin sensitivity 7-9. GPR43 is coupled to the G_i/o_ or G_q_ signalling pathways that transduce the downstream signalling cascades. Previous studies demonstrated that GPR43 is expressed at a high level in the adipose, immune, gastrointestinal endocrine and pancreatic beta cells. The activation of GPR43 is involved in adipocyte differentiation, adipogenesis 10, neutrophil chemotaxis and insulin secretion 11, suggesting that GPR43 activation may have various metabolic effects. However, possible roles of GPR43 in podocyte injury and insulin resistance in DN have not been studied.

Adenosine 5'-monophosphate (AMP)-activated protein kinase (AMPK) is an energy sensor that maintains energy homeostasis under cellular stress conditions 12, 13. Mammalian AMPK is a heterotrimeric enzyme consisting of the catalytic α subunit, regulatory β subunit and targeting γ subunit. Phosphorylation of Thr172 on the AMPK α subunit is essential for covalent AMPK activation and enzyme activity that has significant effects on target gene expression 13. The activity of AMPK is extensively regulated by the level of AMP and multiple upstream signals. AMPK positively regulates insulin-dependent glucose uptake and insulin signalling 14-16. However, the AMPK activity is inhibited in disorders associated with insulin resistance 17, 18.

Our previous study demonstrated that the dysbiosis of gut microbiota contributed to the tubulointerstitial injury of DN through the disruption of cholesterol homeostasis 19**.** Therefore, using diabetic animal models, GPR43 gene knockout (KO) mice, and cultured podocytes, this study aimed to investigate the possible roles of GPR43 in podocye injury of early DN and to explore its potential mechanisms involved in gut microbiota, insulin resistance, and the modulation of AMPKα activity.

## Materials and Methods

### Human specimens

The patients with renal biopsy proved DN (at classes II, III and IV of Tervaert's renal pathological classification) 20 were enrolled. The kidney samples of patients with DN were obtained from the Renal Department, Zhong Da Hospital Affiliated to Southeast University, Nanjing, China. Kidney samples of the controls were obtained from the Urology Department, which were from the healthy kidney poles of individuals who underwent tumour nephrectomies and without diabetes or other renal diseases. Clinical characteristics in healthy individuals and patients with DN were summarized in Table 1. The investigation was conducted in accordance with the principles of the Declaration of Helsinki and was approved by the Research Ethics Committee of Southeast University after informed consent was signed by the patients.

### Animal studies

#### Construction of GPR43 gene knockout mice

C57BL/6-background heterozygous GPR43 gene knockout (GPR43 KO) mice were obtained from GemPharmatech (Nanjing, China). Heterozygous N7 intercrossing was performed to produce GPR43 KO mice and wild type littermate control mice. Genotyping was done by using a technology of polymerase chain reaction.

#### Preparation of diabetic models

For diabetic rat model, 8-week-old male Sprague-Dawley rats purchased from Bikai Company (China) were challenged by a single intraperitoneal injection with 100 mg/kg of streptozotocin (STZ) (Sigma, USA). At 72 h after STZ injection, blood glucose levels were measured.

For the diabetic mouse model, 6-week-old GPR43 KO mice were intraperitoneally injected with 50 mg/kg of STZ for 5 consecutive days. Wild type mice received citrate buffer injections and were used as the control (Vehicle). Mice with blood glucose levels higher than 16.7 mmol/L were considered successful induction of diabetes 21. The mice were then divided into four groups: control group (Ctrl) (*n* = 6), GPR43 knockout group (GPR43 KO) (*n* = 6), diabetes group (DM) (*n* = 6), and GPR43 knockout diabetes group (GPR43 KO+DM) (*n* = 7). The body weight in each group of mice was measured every week. Blood glucose in each group of mice was measured randomly every two weeks. The mice were humanely sacrificed at Week 12.

#### Depletion of gut microbiota

To observe the effects of gut microbiota on podocyte insulin resistance in DN, after successful induction of diabetic rat models, depletion of gut microbiota was achieved by an antibiotic cocktail. The antibiotic cocktail consisted of four antibiotics and an antifungal drug (100 mg/kg of ampicillin, 25 mg/kg of vancomycin, 100 mg/kg of metronidazole, 100 mg/kg of neomycin, and 10 mg/kg of amphotericin B, Sigma). The antibiotic cocktail was made fresh every 48h 22. The rats were divided into three groups and maintained for 8 weeks: wild type control rats (Ctrl), diabetic rats treated with vehicle (DM), and diabetic rats treated with daily oral gavage of antibiotics cocktail (DM+AB). Distribution of gut microbiota was analysed by 16S ribosomal DNA sequencing.

#### Faecal microbiota transplantation

Faecal microbiota transplantation (FMT) is a strategy to evaluate the influence of gut microbiota. One week before transplantation, sufficient faeces from wild type rats were collected, weighed, placed in a pre-cooled sterile phosphate buffered saline (PBS) solution containing 10% glycerol, and homogenized into a suspension. The suspension was filtered through a sterile stainless steel filter with a pore diameter of 0.2 μm and stored at -80 °C 23. After successful induction of diabetic rat models, rats were randomly divided into the control group (Ctrl), diabetes group (DM), and diabetes with faecal microbiota transplantation group (DM+FMT). Each faecal sample (100 mg) was suspended in 1.0 mL sterile PBS under anaerobic condition. The DM+FMT group was administered with 200 μL of the suspended faecal microbiota by oral gavage. During the transplantation, the suspension was thawed, mixed, and slowly infused into the stomach of diabetic rats through a catheter. The DM group was given sterile PBS solution containing 10% glycerol. After transplantation, the distribution of gut microbiota was analysed by 16S ribosomal DNA sequencing.

All protocols of animal experiments were performed in accordance with the guidelines of the Institutional Animal Care and Use Committee of the Southeast University.

### Cell Culture

Immortalized mouse podocytes were a generous gift of Professor Ai Hua Zhang from Children's Hospital of Nanjing Medical University; the cells were cultured in RPMI 1640 medium with 10% foetal bovine serum (FBS) at 33 °C as described previously 24. Podocytes were thermo-shifted to 37 °C for 10 days before they were used in the experiments. Chemical agents, GLPG0974 (10 μmol/L) (R&D Systems, USA), AICAR (10 μmol/L), U73122 (10 μmol/L), and Go 6983 (10 μmol/L) (Selleck, USA), were acquired for the experiments.

### The 16S ribosomal DNA sequencing

Fresh faecal samples at certain time points were collected and frozen at -80 °C until DNA extraction. The faecal pellets were then resuspended in PBS and DNA was extracted using a QIAamp DNA stool mini kit (Qiagen, Germany). Microbiota profiling was performed using high-throughput 16S ribosomal DNA amplicon sequencing corresponding to the hypervariable V3-V4 region.

### Biochemical assays

Blood samples were used for biochemical assays of fast glucose, blood urea nitrogen (BUN) and serum creatinine (Scr).

### High-performance liquid chromatography (HPLC)

Briefly, serum samples were acidified with 25% metaphosphoric acid (1 volume of acid and 5 volumes of the sample) for 30min on ice. After acidification, samples were then centrifuged at 12,000 × g for 15min at 4 °C, and the supernatants were filtered and stored at -80 °C until HPLC analysis. The concentrations of acetate in the serum of the rats were analysed by an HPLC Ultimate 3000 system (Dionex, USA).

### Urinary albumin to creatinine ratio (ACR)

Urinary albumin level was measured by a mouse microalbuminuria enzyme-linked immunosorbent assay (ELISA) kit (Elabscience, China), and urinary creatinine was determined by an assay based on the Jaffe method (Jiancheng, China).

### Periodic acid-Schiff (PAS) staining

Renal cortical tissue samples were fixed and then embedded in paraffin. The histologic features were assessed by PAS using standardized protocols. Histological images were visualized using a light microscope at ×40 magnification and analysed using the Image J software. The staining was quantified in positive area per glomerulus area. Twenty glomeruli per section were analysed for glomerular mesangial expansion by semi-quantitative analysis 25.

### Immunofluorescent staining

Immunofluorescent staining of paraffin-embedded kidney sections was performed to determine the expression levels of WT-1, GPR43 and p-Akt. A donkey anti-mouse or goat anti-rabbit IgG antibody conjugated to fluorescein (1:500, Jackson immunoresearch, USA) was used as a secondary antibody, and the slides were counterstained the 4, 6-diamidino-2-phenylindole (DAPI). The fluorescent images were visualized under a confocal microscopy system (Olympus, Japan). Fluorescence intensity was analysed with the Image J software. WT-1 is a specific biomarker of podocytes. Immunofluorescent staining of WT-1 was used to assess the number of podocytes in glomeruli. WT-1-positive nuclei were statistically counted in 20 glomeruli per sample by two blinded investigators 26.

### Transmission electron microscopy (TEM)

Morphological changes of the kidney ultrastructure were evaluated by TEM (Hitachi, Japan). The protocol of sample processing was described in our previous study 19.

### Western blotting

The renal cortex tissues from the diabetic models or podocytes were lysed on ice for 30s with a RIPA lysis buffer. Equal protein concentrations were loaded using sodium dodecyl sulphate (SDS) -polyacrylamide gel electrophoresis and transferred to an immobilon-P polyvinylidene difluoride (PVDF) membrane (Millipore, USA). After blocking by 5% BSA solution, the membranes were then incubated overnight at 4 °C with primary antibodies against nephrin and fibronectin (1:500 dilution, Santa Cruz, USA), collagen I (1:1000 dilution, Abcam, USA), α-SMA (1:1000 dilution, Abcam, UK), GPR43 (1:1000 dilution, Merck, Germany), AMPKα (1:1000 dilution, Cell Signaling, USA), pAMPKα (Thr172) (1:1000 dilution, Cell Signaling, USA), Akt (1:1000 dilution, Cell Signaling, USA), pAkt (Ser473) (1:1000 dilution, Cell Signaling, USA), PKC (1:500 dilution, Santa Cruz, USA), pPKC (1:500 dilution, Santa Cruz, USA), PLCγ1 (1:1000 dilution, Abcam, UK), pPLCγ1 (1:1000 dilution, Abcam, UK) and β-actin (1:3000 dilution, KeyGEN, Netherlands) followed by horseradish peroxidase (HRP)-conjugated goat anti-mouse or anti-rabbit IgG for 1h. Quantification was performed by measuring the intensity with the ImageJ software.

### Real-time PCR

RNA was extracted using TRIzol reagent (Invitrogen, USA). Total RNA was added to a reaction system and assayed using a PrimeScript RT master mix (Vazyme, China). mRNA was quantified using SYBR Green master mix (Vazyme, China) by a 7300 real-time PCR detection system (Applied Biosystems, USA). β-actin served as the internal normalization gene. The relative expression of the target genes was calculated as ΔΔCt. The mouse gene primers were summarized in Table 2.

### GPR43 siRNAs transfection

Scrambled siRNA and GPR43 siRNA (GenePharma, China) were mixed with Lipofectamine 2000 (Invitrogen, USA) according to the manufacturer's instructions. The sequences of the siRNAs were as follows: GPR43, 5'-GGCACUGAGAACCAAAUAATT-3'; and nonspecific scrambled siRNA, 5'-UUCUCCGAACGUGUCACGUTT-3'.

### Glucose uptake assay

The glucose uptake assay in podocytes was performed as described previously 27. In brief, after 12 h serum starvation, cells were stimulated with 100 nmol/L insulin for 30min in Krebs-Ringer HEPES buffer at 37 °C. 2-Deoxy-2-[(7-nitro-2,1,3-benzoxadiazol-4-yl) amino]-D-glucose (2-NBDG) (500 nmol/L) was then added to the cells. Cells were washed twice with ice-cold PBS After incubation. The uptake of 2-NBDG was evaluated by detecting the changes in the fluorescence intensity in the cells. The optical density value was read after an enzyme-linked fluorescent immunoassay at the wavelength of 490 nm. Each group of the cells was incubated with cell counting kit-8 (CCK-8) for 4h, and the errors caused by the differences in the cell numbers in each group in the fluorescence intensity assay were corrected versus the CCK-8 signal.

### Statistical analysis

Statistical analysis was performed using the GraphPad Prism version 7.0 (GraphPad Software Inc., USA). Values were expressed as the mean ± standard deviation (SD). Two groups of the data were compared using the two-tailed Student's t test. Data from more than two groups were compared using one-way analysis of variance (ANOVA) followed by Turkey's post-test. *P* values were adjusted for multiple comparisons when appropriate. *P* < 0.05 was considered to indicate statistical significance.

## Results

### Increased GPR43 expression in the kidneys of DN was involved in podocyte insulin resistance

**Figure 1:**
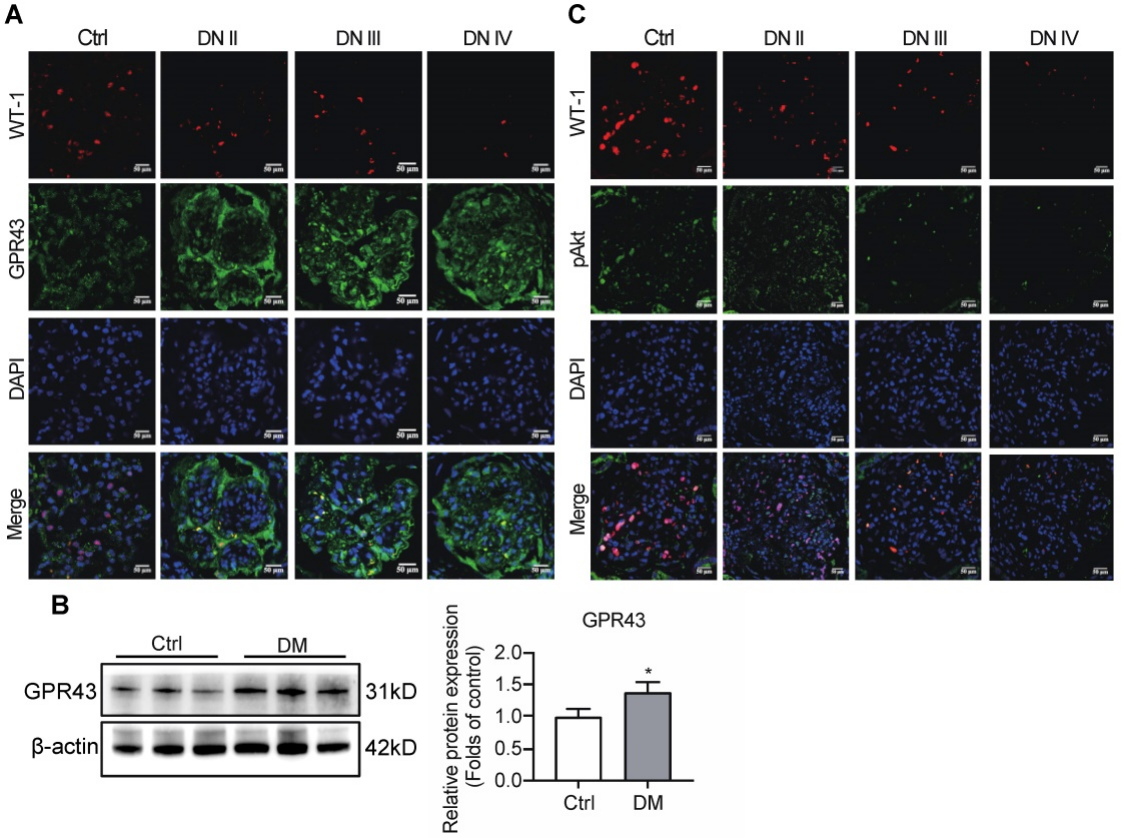
** Diabetes mellitus enhances the expression of GPR43 in the kidney. (A)** Representative confocal immunofluorescent images illustrating an increase in the expression of GPR43 in the kidneys of the patients with DN of various degrees of renal biopsy-proved pathological damage. Scale bar, 50 µm. **(B)** Western blotting analysis of expression of GPR43 protein in the renal cortex of vehicle- or streptozotocin (STZ)-treated diabetic mice at Week 12 (mean ± SD, *P < 0.05 vs. Ctrl, n = 3). **(C)** Representative confocal immunofluorescent images illustrating the expression of phosphorylated Akt and WT-1 in the kidney of healthy controls and the patients with DN of various degrees of renal biopsy-proved pathological damage. Scale bar, 50 µm.

To observe the effects of GPR43 on the regulation of insulin signalling in podocytes, the expression of GPR43 in the kidneys was assayed in patients with various stages of biopsy-proved DN and in diabetic mice. Immunofluorescent staining demonstrated that the GPR43 protein expression was increased in podocytes of DN patients (Figure 1A) and in the kidneys of diabetic mice according to Western blotting (Figure 1B). We then evaluated the Akt protein phosphorylation levels of podocytes in human biopsy samples of the diabetic kidney using immunofluorescent staining. The results showed that co-localization of WT-1 with phosphorylated Akt protein was gradually decreased in the kidneys at various stages of DN in patients (Figure 1C), suggesting that podocyte insulin resistance is deteriorating during the development of renal injury in DN. Therefore, we assumed that increased GPR43 expression can be closely associated with a decrease in podocyte insulin resistance.

### Acetate downregulated insulin signalling in podocytes via the activation of GPR43

**Figure 2:**
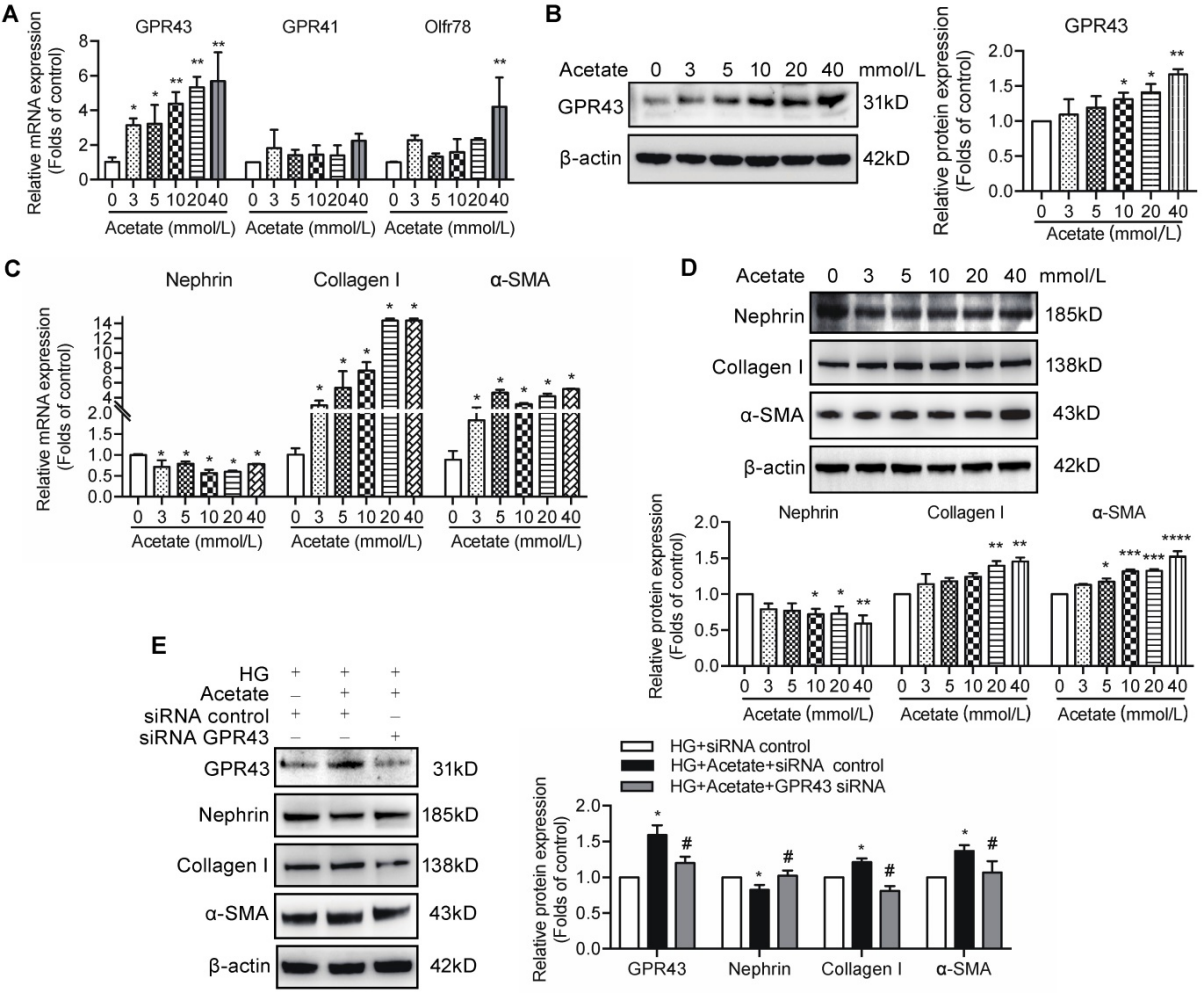
** The effects of acetate-induced GPR43 activation on podocyte injury. (A)** Real-time PCR analysis of the mRNA levels of GPR43, GPR41, and Oflr78 in podocytes, which were treated with different concentrations of acetate (3, 5, 10, 20 and 40 mmol/L) under the high glucose condition (30 mmol/L D-glucose, HG) for 24h (mean ± SD, ^*^P < 0.05, ^**^P < 0.01 *vs*. Ctrl,* n* = 3). **(B)** Western blotting analysis of the protein levels of GPR43 in podocytes, which were treated with different concentrations of acetate (3, 5, 10, 20 and 40 mmol/L) under the HG condition for 24h (mean ± SD, ^*^P < 0.05 *vs*. Ctrl,* n* = 3). **(C)** Real-time PCR analysis of the mRNA levels of nephrin, collagen I and α-SMA in podocytes, which were treated with different concentrations of acetate (3, 5, 10, 20 and 40 mmol/L) under the HG condition for 24h (mean ± SD, ^*^P < 0.05 *vs*. Ctrl,* n* = 3). **(D)** Western blotting analysis of the protein levels of nephrin, collagen I and α-SMA in podocytes, which were treated with different concentrations of acetate (3, 5, 10, 20 and 40 mmol/L) under the HG condition for 24h (mean ± SD, ^*^P < 0.05,^ **^P < 0.01, ^***^P < 0.001 *vs*. Ctrl,* n* = 3). **(E)** Western blotting analysis of the protein level of nephrin, collagen I and α-SMA in podocytes (mean ± SD, ^*^P < 0.05 *vs*. HG+ siRNA control, *^#^P* < 0.05 *vs*. HG + acetate + siRNA control,* n* = 3).

Short-chain fatty acids (SCFAs), especially acetate, are known activators of GPR43. To evaluate the role of acetate in GPR43 activation, we determined the effects of various different concentrations of acetate on the GPR43 gene and protein expression in podocytes in the absence or presence of high concentration of glucose. The results showed that acetate remarkably increased the mRNA and protein expression levels of GPR43 in podocytes in a dose-dependent manner (Figure 2A, 2B). Acetate at the concentration of 40 mmol/L also activated Oflr78 in podocytes; however, an increase in the mRNA levels of Oflr78 was less pronounced compared with that observed in the case of GPR43 (Figure 2A). Acetate stimulation induced phenotypic transformation of podocytes, characterized by dramatically increased α-SMA and collagen I expression levels and decreased nephrin expression at the mRNA and protein levels in podocytes (Figure 2C, 2D). These effects were dramatically attenuated after the treatment with GPR43 siRNA (Figure 2E).

**Figure 3:**
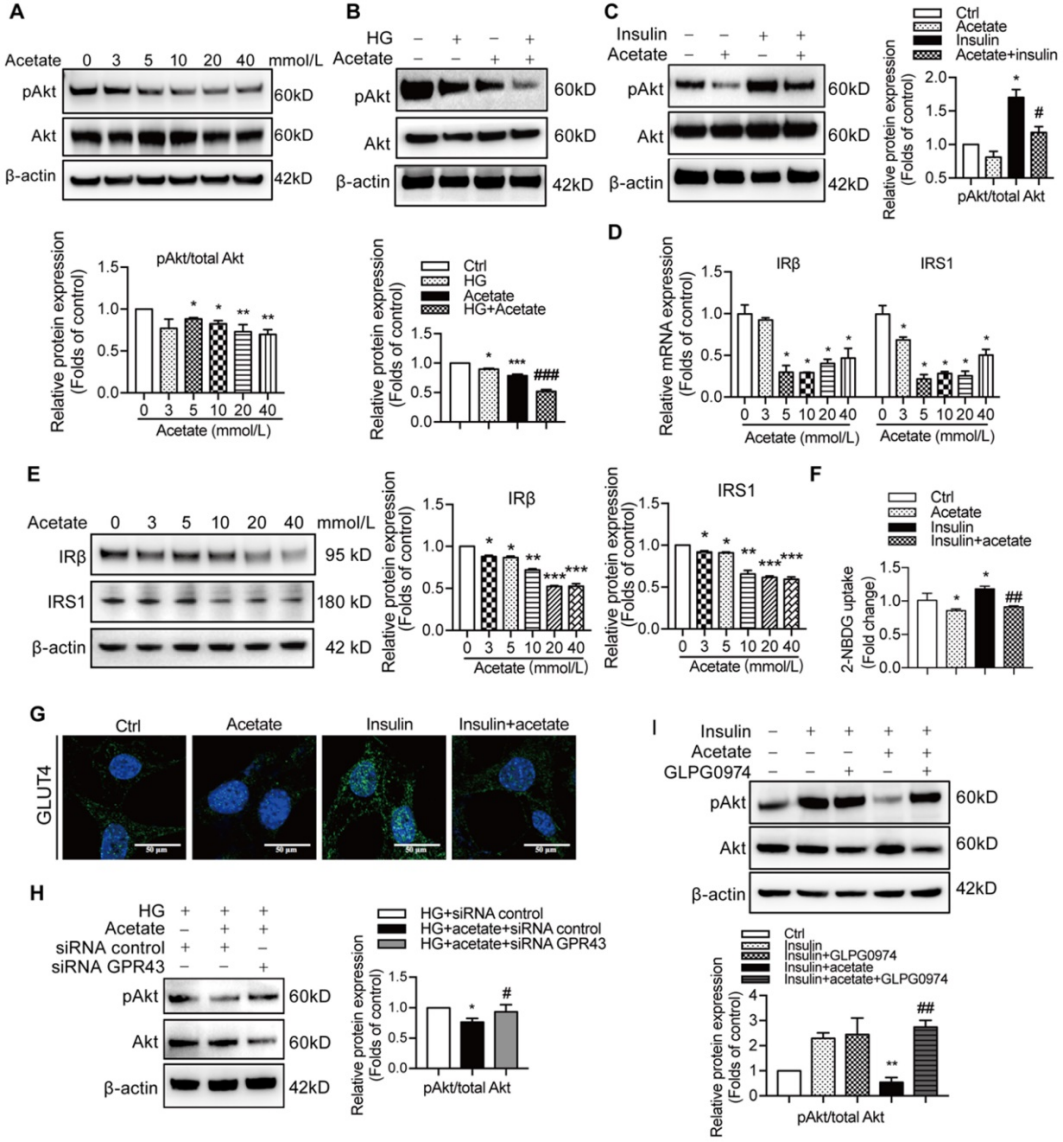
** The activation of GPR43 suppressed insulin signalling in podocytes. (A)** Inhibitory effects of acetate (3, 5, 10, 20 and 40 mmol/L) on Akt phosphorylation in podocytes under the HG condition for 24h. (mean ± SD, ^*^P < 0.05,^ **^P < 0.01 *vs*. Ctrl,* n* = 3). **(B)** Inhibitory effects of acetate (10 mmol/L) on Akt phosphorylation in podocytes (mean ± SD, ^*^P < 0.05 *vs*. Ctrl, ^***^P < 0.001 *vs*. Ctrl, and *^###^P* < 0.001 *vs*. HG,* n* = 3). **(C)** Inhibitory effects of acetate (10 mmol/L) on the suppression of insulin-induced Akt phosphorylation in podocytes (mean ± SD, ^*^P < 0.05 *vs*. Ctrl, *^#^P* < 0.05 *vs*. Insulin,* n* = 3). **(D)** Inhibitory effects of acetate (3, 5, 10, 20 and 40 mmol/L) on the suppression of the IRβ and IRS1 mRNA expression in podocytes under the HG condition for 24h (mean ± SD,^*^P < 0.05 *vs*. Ctrl,* n* = 3). IRβ, insulin receptor β; IRS1, insulin receptor substrate 1. **(E)** Inhibitory effects of acetate (3, 5, 10, 20 and 40 mmol/L) on the suppression of IRβ and IRS1 protein expression in podocytes under the HG condition for 24h (mean ± SD,^*^P < 0.05, ^**^P < 0.01, ^***^P < 0.001 *vs*. Ctrl,* n* = 3). **(F)** Podocytes significantly increased 2-NBDG uptake in response to insulin, and acetate (10 mmol/L) selectively reduced 2-NBDG uptake (mean ± SD, ^*^P < 0.05 *vs*. Ctrl, *^##^P* < 0.01 *vs*. Insulin,* n* = 3). **(G)** Representative confocal immunofluorescent images illustrating the suppression of the insulin-induced translocation of GLUT4 protein to the cellular membrane of podocytes mediated by acetate (GLUT4, Green; DAPI, Blue; original magnification × 600, scale bars, 50 µm). **(H)** Effects of GPR43 knockdown by siRNA on restoration of insulin-induced Akt phosphorylation regulated by acetate (mean ± SD, ^*^P < 0.05 *vs*. HG + siRNA control, *^#^P* < 0.05 *vs*. HG + acetate + siRNA control,* n* = 3). **(I)** Effects of GPR43 inhibition by GLPG0974 on restoration of insulin-induced Akt phosphorylation regulated by acetate (mean ± SD, ^**^P < 0.01 *vs*. HG + siRNA control, *^##^P* < 0.01 *vs*. HG + acetate + siRNA control,* n* = 3).

Preservation of insulin signalling is crucial to protection of podocytes from the injury. Therefore, we measured the effects of GPR43 activation on insulin signalling in podocytes. The results showed that acetate treatment at the concentrations of 3, 5, and 10 mmol/L downregulated the PI3K-Akt signalling in podocytes via a decrease in the Akt protein phosphorylation in the absence or in the presence of high concentration of glucose (Figure 3A, 3B). Furthermore, acetate inhibited the Akt protein phosphorylation in podocytes induced by insulin stimulation (Figure 3C). In addition, acetate inhibited the mRNA and protein expression of insulin receptor substrate 1 (IRS1) and insulin receptor β (IRβ) with or without high concentration of glucose (Figure 3D, 3E). Furthermore, acetate inhibited glucose uptake of podocytes which was stimulated by insulin (Figure 3F), which was correlated with the disruption of the insulin-induced GLUT4 translocation to the cellular membrane mediated by acetate as shown by immunofluorescent staining (Figure 3G). Then, we assessed a possible role of GPR43 in the acetate-mediated downregulation of insulin signalling in podocytes. GPR43 siRNA or GLPG0974, an antagonist of GPR43, were able to override acetate-mediated decrease in Akt protein phosphorylation in podocytes (Figure 3H, 3I). Thus, genetic and pharmacologic approaches indicate that GPR43 is necessary for the downregulation of insulin signalling in response to acetate.

### GPR43 deficiency alleviated insulin resistance-mediated injuries of podocytes and the kidneys in DN

**Figure 4:**
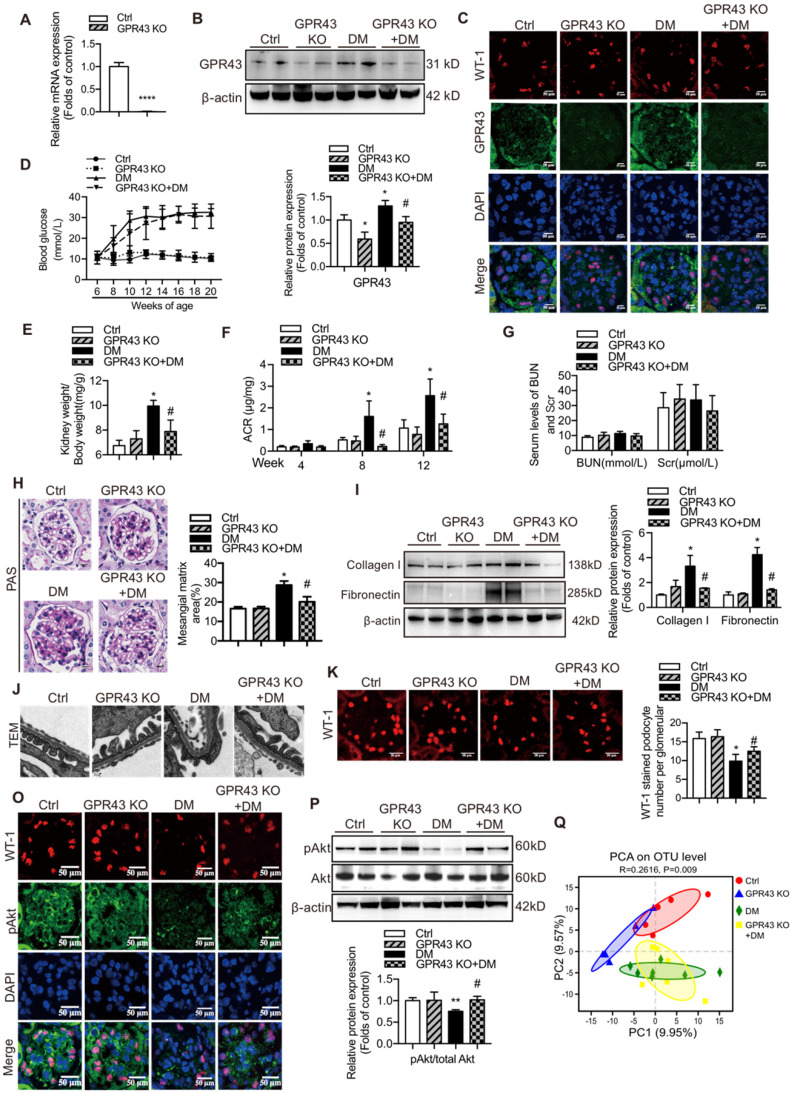
** GPR43 deletion attenuated podocyte injury and albuminuria of diabetic mice. (A)** Real-time PCR of the GPR43 mRNA expression in the pooled renal cortex samples from Ctrl and GPR43 KO mice (means ± SD, ^****^P < 0.0001 *vs.* Ctrl,* n* = 6). **(B)** Representative Western blotting images and densitometric analysis of the GPR43 protein expression in the renal cortex in the four groups of mice (means ± SD, ^*^P < 0.05 *vs.* Ctrl, *^#^P* < 0.05 *vs.* DM). **(C)** Representative confocal microscopic images illustrating the expression of GPR43 and WT-1 in podocytes from the four groups of mice (original magnification × 400, scale bars, 50 µm). **(D)** The levels of random blood glucose of the four groups of mice were taken every two weeks during the study. **(E)** Kidney weight to body weight ratio was elevated in the DM mice compared with that in the Ctrl mice and significantly reduced in the GPR43 KO+DM mice (means ± SD, ^*^P < 0.05 *vs.* Ctrl, *^#^P* < 0.05 *vs.* DM). **(F)** Urinary albumin-to-creatinine ratio (ACR) was detected in spot urine samples of mice collected respectively at Week 4, 8 and 12. ACR was elevated in the DM mice compared to that in the Ctrl mice; however, ACR was significantly reduced in the GPR43 KO+DM mice at Weeks 8 and 12 after the induction of diabetes (means ± SD, ^*^P < 0.05 *vs.* Ctrl, *^#^P* < 0.05 vs. DM). **(G)** Lack of differences in the serum levels of BUN and Scr in four groups of mice. BUN, blood urea nitrogen. Scr, serum creatinine. **(H)** Representative images of PAS-stained kidney sections of all groups of mice (original magnification ×400, scale bars, 50 µm). Bar graph analysis of the semi-quantification estimate of the mesangial expansion score (means ± SD, ^*^P < 0.05 *vs*. Ctrl, *^#^P* < 0.05 *vs.* DM). **(I)** Representative Western blotting images and densitometric analysis of collagen I and fibronectin in the renal cortex samples of all groups of mice (means ± SD, ^*^P < 0.05 *vs.* Ctrl, *^#^P* < 0.05 *vs.* DM). **(J)** Representative images and estimation of ultrastructural changes in podocyte morphology, including podocyte effacement and glomerular basement membrane (GBM) thickness, observed by electron microscopy (original magnification × 40,000, scale bars, 500 nm). **(K)** Representative confocal immunofluorescent images of WT-1 staining in the kidney sections of all groups of mice (original magnification × 400, scale bars, 50 µm). The WT-1-positive nuclei were statistically counted (means ± SD, ^*^P < 0.05 *vs.* Ctrl, *^#^P* < 0.05* vs.* DM). **(L)** Representative confocal microscopic images showing the expression of pAkt in podocytes from various groups of mice (original magnification × 400, scale bars, 50 µm). **(M)** Representative Western blotting images of the pAkt and total Akt protein expression in the renal cortex samples of the four groups of mice (means ± SD, ^**^P < 0.01 *vs.* Ctrl, *^#^P* < 0.05 *vs.* DM). **(N)** Principal component analysis (PCA) of distances between the communities of the faecal microbiota in the four groups of mice.

Considering strong association of GPR43 activation with insulin signalling in podocytes, we estimated functional relevance of GPR43 to the kidney injury in DN by using genetic knockout approaches *in vivo*. We generated GPR43 KO mice based on the CRISPR/Cas9 system. The results of real-time PCR confirmed successful construction of the GPR43-KO model (Figure 4A). The protein expression levels of GPR43 were significantly decreased in the kidney of GPR43 KO+DM mice compared with that in the DM group (Figure 4B, 4C). There were no differences in the blood glucose level (Figure 4D) and kidney weight to body weight ratio (Figure 4E) between the controls and the GPR43 KO mice. Although the blood glucose levels in the DM and DM+GPR43 KO mouse groups were no differences (Figure 4D), the ratio of kidney weight to body weight in the DM+GPR43 KO mice was decreased compared with that in the DM group (Figure 4E). Urinary albumin excretion rates at Week 8 and Week 12 were decreased in the GPR43 KO+DM group compared with that in the DM group (Figure 4F). However, there were no differences in the levels of BUN and Scr among the four groups (Figure 4G). Therefore, we subsequently evaluated the role of GPR43 KO in the pathological renal injury. The PAS staining showed that mesangial expansion was significantly decreased in the GPR43 KO+DM mice compared with that in the DM mice (Figure 4H), which was in accordance with a decrease in the protein expression levels of collagen I and fibronectin (Figure 4I). These data suggested a potential protective effect of GPR43 deletion on the development of DN. Moreover, electron microscopy observation demonstrated that there was a significant improvement in podocyte foot process effacement with reduced thickness of the glomerular basement membrane (GBM) in the diabetic GPR43 KO mice compared with that in the DM mice (Figure 4J). The density of podocytes was further assessed by immunofluorescent staining of Wilms' tumor-1 (WT-1), a specific biomarker of podocytes. The results showed that podocyte number was decreased in the kidneys of the DM group. However, podocyte density was increased in the kidneys of diabetic mice with GPR43 knockout (Figure 4K). Thus, these data suggest that GPR43 deletion protects podocytes from injury in DN.

To observe the effects of GPR43 activation on the insulin signalling in the kidneys of diabetic mice, we assayed Akt protein phosphorylation (Ser473) in podocytes by Western blotting and immunofluorescent staining. The results indicated that GPR43 KO increased Akt protein phosphorylation in podocytes in diabetic mice (Figure 4L, 4M), suggesting that GPR43 depletion ameliorated the insulin signalling in podocytes with the hyperglycaemia background.

SCFAs are known to be mainly produced by gut microbiota. Thus, we assessed a possible role of gut microbiota in the acetate/GPR43 pathway activation-mediated impairment of insulin signalling in podocytes. The results showed that gut microbiota had significantly different composition and distribution in the DM group versus the controls; these characteristics were not substantially influenced by GPR43 KO as illustrated by the principal component analysis (Figure 4N). This result suggests that gut microbiota may be involved in the activation of GPR43.

### Gut microbiota activated GPR43 via released acetate to contribute to the development of DN

**Figure 5:**
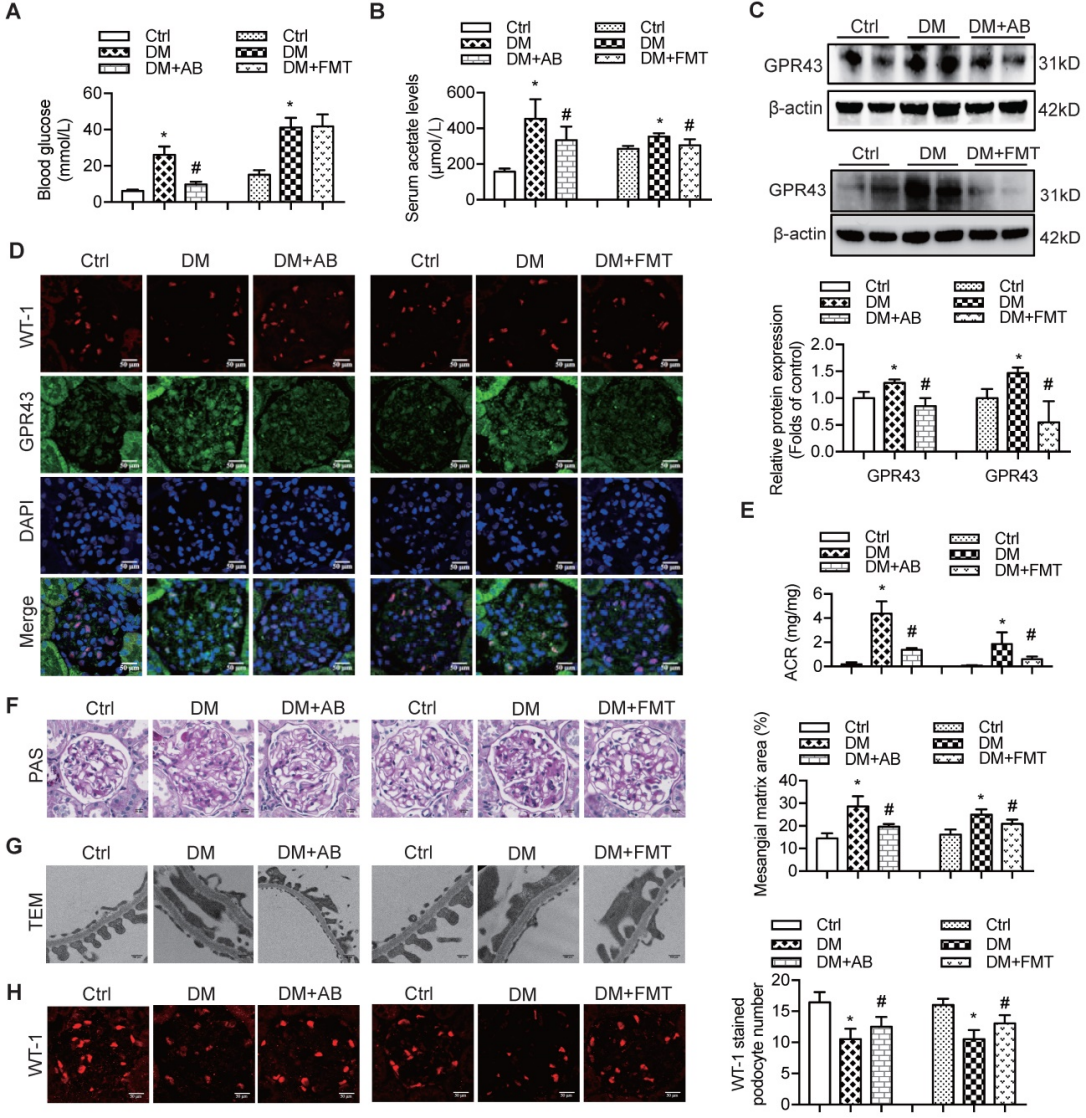

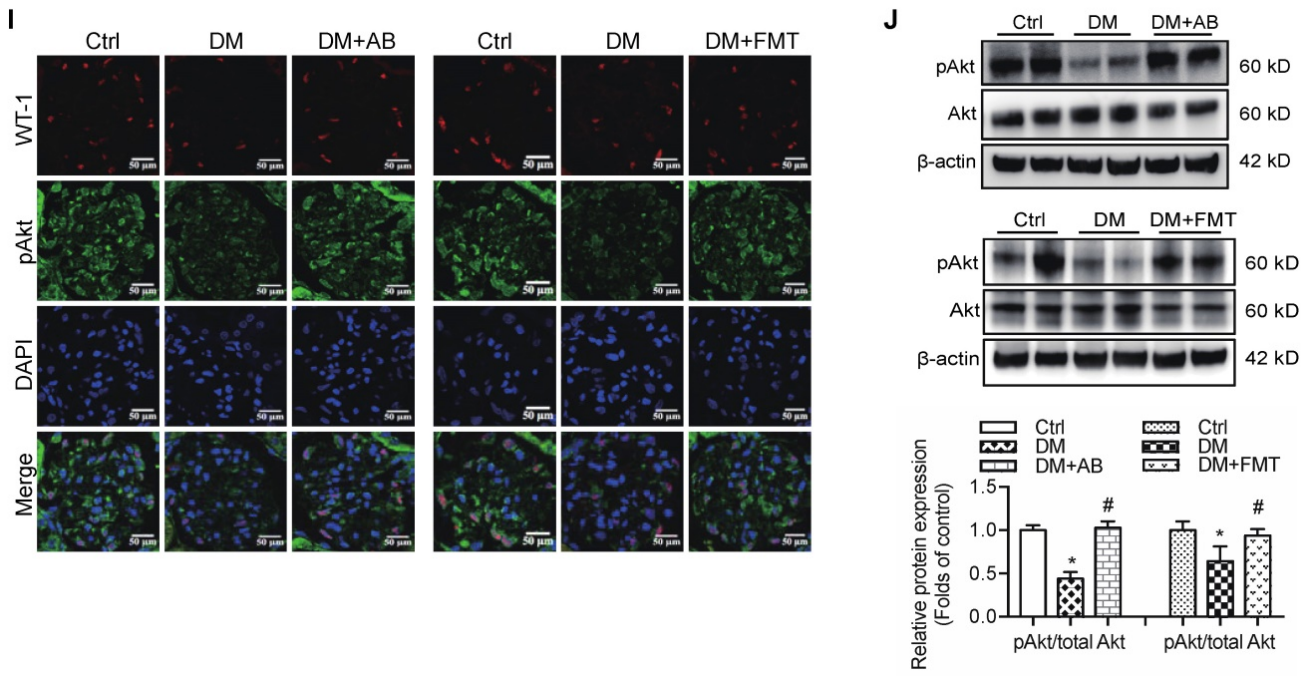
** The treatment with broad-spectrum antibiotics or faecal microbiota transplantation (FMT) improved glomerular injury in STZ-induced diabetic rats. (A)** Serum level of glucose was elevated in DM rats compared to that in Ctrl rats significantly reduced in DM+AB rats (means ± SD, ^*^P < 0.05 *vs.* Ctrl, *^#^P* < 0.05 *vs.* DM). **(B)** Serum level of acetate was elevated in DM rats compared to that in the Ctrl rats and significantly reduced after the treatment with antibiotics or FMT (means ± SD, ^*^P < 0.05 *vs.* Ctrl, *^#^P* < 0.05 *vs.* DM). **(C)** Representative Western blotting images showing that the treatment with antibiotics or FMT reduced the expression of GPR43 in the kidney of DM rats (means ± SD, ^*^P < 0.05* vs.* Ctrl, *^#^P* < 0.05 *vs.* DM). **(D)** Representative confocal microscopic images showing the expression of GPR43 in podocytes from various groups of rats (original magnification × 400, scale bars, 50 µm). **(E)** Urinary albumin to creatinine ratio (ACR) was elevated in DM rats compared with that in the Ctrl rats and significantly reduced after the treatment with antibiotics or FMT (means ± SD, ^*^P < 0.05 *vs.* Ctrl, *^#^P* < 0.05 *vs.* DM). **(F)** Representative images of the PAS-stained kidney sections of all groups of rats (original magnification × 400, scale bars, 50 µm). Bar graphs showing semi-quantitative estimation of the mesangial expansion score (means ± SD,^ *^P < 0.05 *vs.* Ctrl, *^#^P* < 0.05 *vs.* DM). **(G)** Representative photomicrographs of the glomerular basement membrane (GBM) thickness and the degree of foot processes in various groups of rats assayed by transmission electron microscopy (TEM) analysis (original magnification ×40,000, scale bars, 500 nm). **(H)** Representative confocal immunofluorescent images of WT-1 staining in the kidney sections in all groups of rats (original magnification ×400, scale bars, 50 µm). The WT-1-positive nuclei were statistically counted (means ± SD, ^*^P < 0.05* vs.* Ctrl, *^#^P* < 0.05* vs.* DM). **(I)** Representative confocal microscopic images showing the expression of pAkt in podocytes from various groups of rats (original magnification ×400, scale bars, 50 µm). **(J)** Representative Western blotting images and densitometric analysis of pAkt and total Akt proteins in the renal cortex samples of all groups of rats (means ± SD, ^*^P < 0.05* vs.* Ctrl, *^#^P* < 0.05 *vs.* DM).

Therefore, to confirm the modulatory role of gut microbiota and its metabolites in podocyte insulin resistance, broad-spectrum antibiotics or FMT was administered to diabetic rats to induce the depletion of gut microbiota or improvement of gut microbiota dysbiosis. FMT was performed by oral transfer of the gut microbiota extracted from the faeces of healthy donor controls to diabetic rats. Initially, we assayed the serum levels of glucose in rats. The results showed that the serum levels of glucose in the antibiotics-treated diabetic rats were decreased compared to that in diabetic rats. However, there were no differences after the FMT treatment (Figure 5A). Interestingly, the serum level of acetate was increased in diabetic rats compared to that in the controls (Figure 5B), whereas antibiotics or FMT treatment decreased the serum acetate levels (Figure 5B). These results suggest that an increase in serum acetate can be produced by gut microbiota, which mediated the activation of GPR43 in the kidney. Moreover, antibiotic or FMT treatments of diabetic rats decreased the GPR43 protein expression in the kidney (Figure 5C) or podocytes (Figure 5D), accompanied with a decrease in urinary albumin protein excretion (Figure 5E), mesangial matrix expansion (Figure 5F), podocyte foot process effacement (Figure 5G), and increased podocyte number (Figure 5H). Moreover, antibiotics or FMT treatments restored the podocyte insulin signalling in the kidneys of diabetic rats via an increase in Akt phosphorylation as demonstrated by the immunofluorescent co-staining and Western blotting (Figure 5I, 5J). Thus, these findings suggest that dysbiosis of gut microbiota in DN may be involved in the impairment of insulin signalling in podocytes via microbiota metabolites.

### Activation of GPR43 mediated podocyte insulin resistance in DN via inhibition of the AMPKα activity

**Figure 6:**
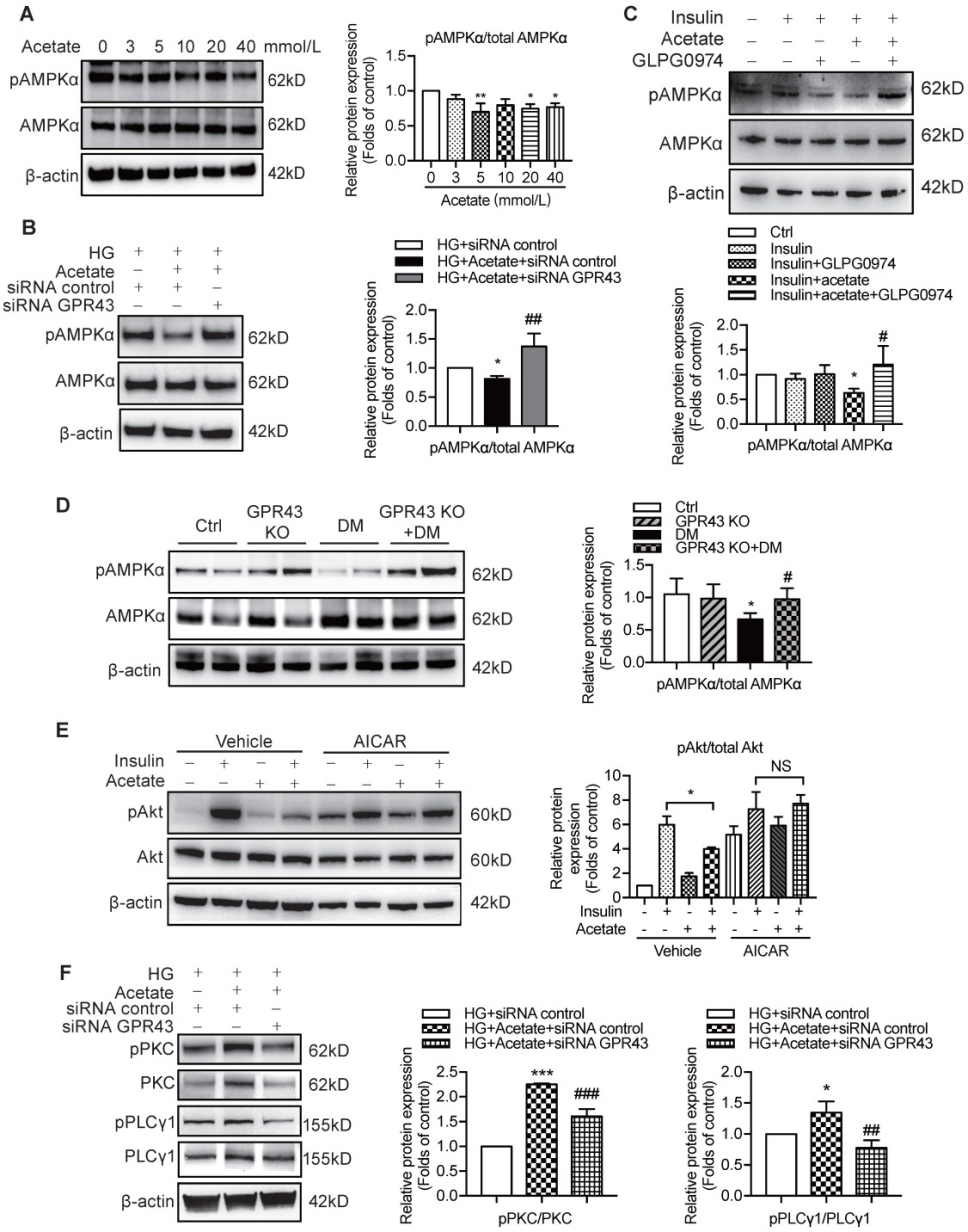

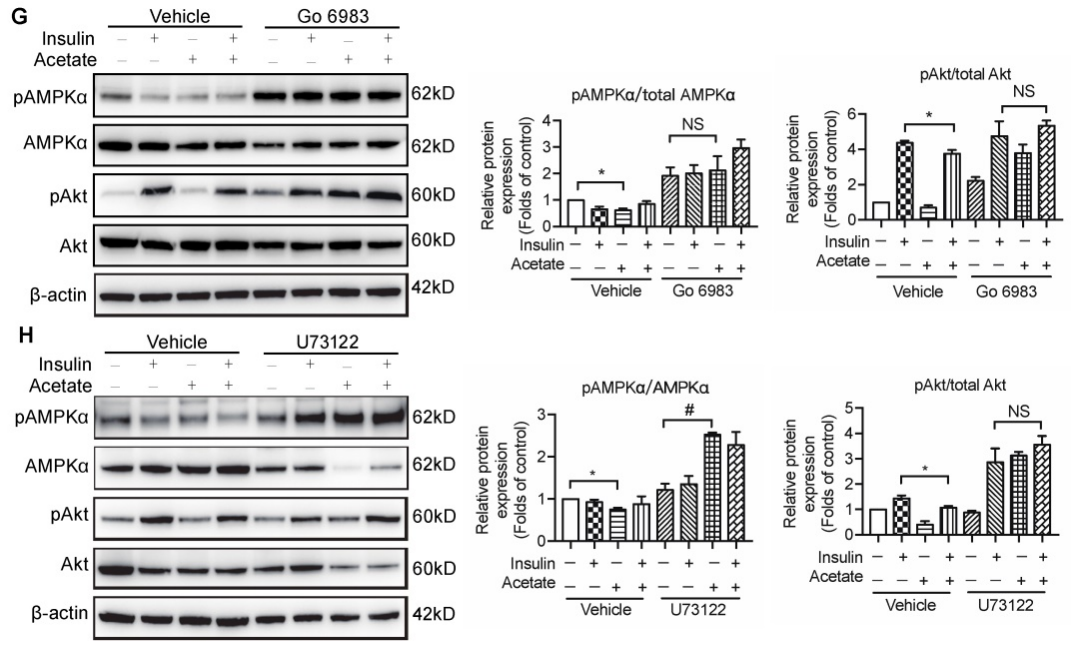
** The activation of GPR43 suppressed insulin signalling via downregulation of AMPKα activity. (A)** Inhibitory effects of acetate on AMPKα phosphorylation in podocytes which were treated with different concentrations of acetate (3, 5, 10, 20 and 40 mmol/L) under the HG condition for 24h (means ± SD, ^*^P < 0.05, ^**^P < 0.01 *vs.* Ctrl, *n* = 3). **(B)** Podocytes were transfected with GPR43 siRNA for 12h and then stimulated with HG or HG plus acetate for 24h. The rescue effects of the GPR43 knockdown on AMPKα phosphorylation which was inhibited by acetate (means ± SD, ^*^P < 0.05 *vs.* HG+ siRNA control, *^##^P* < 0.01 *vs.* HG+acetate+ siRNA control, *n* = 3). **(C)** Podocytes were pretreated with a GPR43 inhibitor (GLPG0974, 10 µmol/L) for 12h and then stimulated with HG or HG plus acetate for 24h. The rescue effects of GPR43 inhibition on AMPKα phosphorylation which was inhibited by acetate (means ± SD, ^*^P < 0.05 *vs.* Insulin, *^#^P* < 0.05 *vs.* Insulin + acetate, *n* = 3). **(D)** Western blotting analysis of AMPKα phosphorylation in renal tissue samples from the four groups of mice (Ctrl, GPR43 KO, DM and GPR43 KO+DM) (means ± SD, ^*^P < 0.05* vs.* Ctrl, *^#^P* < 0.05* vs.* DM). **(E)** Effects of the activation AMPKα signalling mediated by AICAR (10 µmol/L) on restoration of insulin-induced Akt phosphorylation inhibited by acetate (means ± SD, ^*^P < 0.05 *vs.* insulin, *n* = 3). **(F)** The effects of GPR43 knockdown on PKC and PLCγ1 phosphorylation in podocytes which was induced by acetate (mean ± SD, ^*^P<0.05,^ ***^P<0.001 *vs*. HG + siRNA control, *^##^P* < 0.01,*^ ###^P* < 0.001 *vs*. HG + acetate + siRNA control,* n* = 3). **(G)** Effects of PKC signalling inhibition by Go 6983 (10 µmol/L) on restoration of Akt and AMPKα phosphorylation in podocytes treated by acetate (means ± SD, ^*^P < 0.05 *vs.* Insulin; NS, not significant; *n* = 3). **(H)** Effects of PLC signalling inhibition mediated by U73122 (10 µmol/L) on restoration of Akt and AMPKα phosphorylation in podocytes treated by acetate (means ± SD, ^*^P < 0.05 *vs.* Insulin; NS, not significant, *n* = 3).

Previous studies demonstrated that AMPKα functions as a positive regulator of insulin signalling and triggers phosphorylation of Akt at ser473 28. Therefore, we evaluated the effect of the AMPKα activity reduction in diabetic kidney on insulin signalling in podocytes. The results showed that acetate inhibited AMPKα phosphorylation in podocytes in a dose-dependent manner (Figure 6A). However, the acetate-mediated inhibitory effect on AMPKα phosphorylation was overridden by GPR43 siRNA (Figure 6B) and GLPG0974, an inhibitor of GPR43 (Figure 6C). To confirm whether there is a causative link between GPR43 upregulation and decreased AMPKα activity under diabetic condition, we measured AMPKα phosphorylation levels in the kidney of diabetic mice with or without GPR43 KO. As shown in Figure 6D, the level of AMPKα phosphorylation in the kidney of GPR43 KO diabetic mice was increased compared with that in diabetic mice. This result suggests that GPR43 can be a key regulator of inhibition of AMPKα phosphorylation. To clarify the role of AMPKα in activation of GPR43-mediated podocyte insulin resistance, we determined the effect of AMPKα activity restoration induced by an AMPK agonist (5-aminoimidazole-4-carboxamide ribonucleotide, AICAR) on podocyte insulin resistance mediated by GPR43 activation. As predicted, the AICAR treatment reversed the acetate-mediated inhibition of Akt phosphorylation in podocytes (Figure 6E).

To determine how acetate-mediated GPR43 activation is transmitted to the cytoplasm and subsequently activates nuclear/mitochondrial localized AMPKα, we assayed the G protein-coupled pathway of protein kinase C (PKC)-phospholipase C (PLC) in podocytes. As shown in Figure 6F, GPR43 knockdown by siRNA inhibited the PKC and PLCγ1 phosphorylation which was upregulated by acetate. Moreover, PKC inhibitor Go 6983 (Figure 6G) and PLC inhibitor U73122 (Figure 6H) effectively blocked the GPR43-mediated inhibition of Akt phosphorylation in podocytes with or without insulin. These findings suggest that acetate-mediated activation of GPR43 suppresses the bioactivity of insulin in podocytes via the suppression of Akt phosphorylation mediated by the PKC-PLC-AMPKα pathway.

## Discussion

Our study demonstrated that GPR43, a receptor of SCFAs, plays an important role in the modulation of insulin signalling in podocytes. These findings suggest a model of GPR43 influence on podocyte function and survival; excessive expression of GPR43 may impair Akt phosphorylation, thereby inducing podocyte insulin resistance and contributing to the development of DN.

Insulin is crucial for the regulation of glucose trafficking and cytoskeletal contractility in podocytes due to highly expression of insulin receptor and insulin receptor substrate-1. Under physiological conditions, an increase in insulin secretion increases GLUT4-mediated glucose uptake and induces adaptive changes in podocytes 29. The PI3K/Akt pathway is a major downstream signalling cascade of the insulin pathway. Previous studies demonstrated that impaired insulin-induced PI3K/Akt activation resulted in endoplasmic reticulum stress 30, reduced podocyte viability and dysfunction in obese Zucker rats 31. In this study, we demonstrated that insulin-induced Akt phosphorylation is severely disrupted by activation of GPR43. To clarify the role of GPR43 activation in the modulation of podocyte insulin signalling in DN, we generated GPR43-deficient mice. A global deletion of GPR43 reduced albuminuria, prevented mesangial expansion, and preserved podocyte number in STZ-induced diabetic mice, which may be associated with restoration of the podocyte-specific Akt phosphorylation. These findings suggest that GPR43 can be a potential therapeutic target in DN.

Previous studies have demonstrated that gut microbiota and their metabolites are involved in the energy metabolism and insulin resistance in multiple organs 8, 32. Broad-spectrum antibiotics has been frequently used to explore the potential function of the gut microbiome. antibiotic treatment triggered microbiome depletion characterized by a decrease of the microbiome diversity and the SCFA metabolites. A recent study demonstrated that depletion of the gut microbiota by antibiotic intervention in diabetic mice significantly supported the beneficial effects of adipose-derived stem cells as characterized by reversal of hyperglycaemia and increased insulin output 33. FMT is performed by transferring stool samples from a donor to the gastrointestinal tract of a recipient to improve the spectrum and diversity of the microbiome 34. FMT originating from the lean healthy donors is effective for treating peripheral insulin resistance in obese individuals 35. These studies provided a powerful evidence of the interaction between metabolic disorders and the gut microbiota. Here, we have demonstrated a novel, previously unknown role of SCFAs especially with regards to the effects of acetate on podocyte insulin homeostasis. The serum level of acetate was decreased and glucose metabolism were improved in the diabetic rats treated with broad-spectrum antibiotics or the FMT. Consistently, our data suggest that excessive acetate produced by disrupted gut microbiota can be a powerful stimulator of GPR43 activation, which impairs insulin signalling of podocytes. Subsequent analysis demonstrated that the changes in the composition of gut microbiota are closely associated with excessive acetate-induced GPR43 activation and disruption of podocyte insulin homeostasis. Additional studies are needed to determine the exact mechanisms of interaction of gut microbiota with the host energy metabolism.

Acetate can be transported across the gut epithelium to reach the peripheral tissues. GPR43, as one of the SCFA receptors, is abundantly expressed on a variety of immune cells including neutrophils, eosinophils, monocytes, dendritic cells, Treg cells 36, pancreatic cells 37 and adipocytes 38. GPR43 serves as the major SCFA sensor for regulation of energy metabolism, inflammation and immunity in various organs. The controversial effects of GPR43 activation have been demonstrated in the mouse studies in obesity and insulin resistance 39-41. Bjursell *et al.* reported that GPR43-deficient mice fed with high-fat diet have improved glucose tolerance and increased energy expenditure and are thus protected against the development of obesity unlike the wild type mice 39. In contrast, Kimura* et al.* argued that GPR43-deficient mice fed with normal chow or high-fat diet developed weight gain-dependent glucose intolerance accompanied by increased inflammatory macrophage infiltration of the adipose tissue 40. These contradictions may be accounted for by the downstream activation of various G-protein subunits 41. GPR43 activation mediates the signal transduction by coupling with G_q_ and G_i/o_ subunits. In downstream of GPR43 activation, G_q/11_ increases glucose-induced insulin secretion in mouse islets, whereas G_i/o_ decreases the insulin secretion 42. On the other hand, variability in the genetic background and gene-targeting strategies of GPR43 knockout mice used in various studies may partly contribute to these differences in regulation of metabolism. Our results showed that GPR43, and not GPR41 or Oflr78, is the most important acetate receptor in podocytes. In addition, acetate appears to act via a GPR43 dependent mechanism to induce podocyte insulin resistance. Our results support some conclusions of the previous studies because the GPR43-deficient mice exhibited enhanced systemic insulin sensitivity in the present study. At present, there is no clear consensus on the role of GPR43 in glucose metabolism.

The activation of the G_αq_ signalling cascades is expected to promote podocyte injury since the G_αq_ transgenic mice are characterized with proliferation of glomerular capillary, albuminuria, and reduced podocyte-specific proteins expression 43. GPR43 acts as G protein-coupled receptor and its agonist stimulates phospholipase C (PLC) activation and intracellular calcium influx. Interestingly, acetate induced-GPR43 activation stimulates insulin signalling by the PLC-PKC-dependent mechanism *in vitro*, which was consistent with the activation of the GPR43 pathway reported in a previous study. This discrepancy may be due to differences between the species or to other unknown factors. Thus, our study demonstrated that GPR43 can be coupled to the PKC-PLC pathway, suggesting that antagonists targeting the Gαq signalling can be clinically effective in the improvement of podocyte insulin resistance.

In summary, the GPR43 deletion decreased albuminuria and attenuated podocyte injury in STZ-induced diabetic mice, which was associated with increased insulin sensitivity of podocytes. Acetate stimulated GPR43 activation and inhibited the downstream insulin-induced activation of the Akt signalling in cultured podocytes. The treatments of gut microbiota by antibiotics or FMT effectively downregulated the activation of the GPR43 pathway and protected podocytes from the injury in diabetic rats due to a decrease in the serum acetate levels. These data suggest that downregulation of gut microbiota modulated-GPR43 and GPR43-related insulin resistance represents a new therapeutic avenue in DN.

## Figures and Tables

**Table 1:**
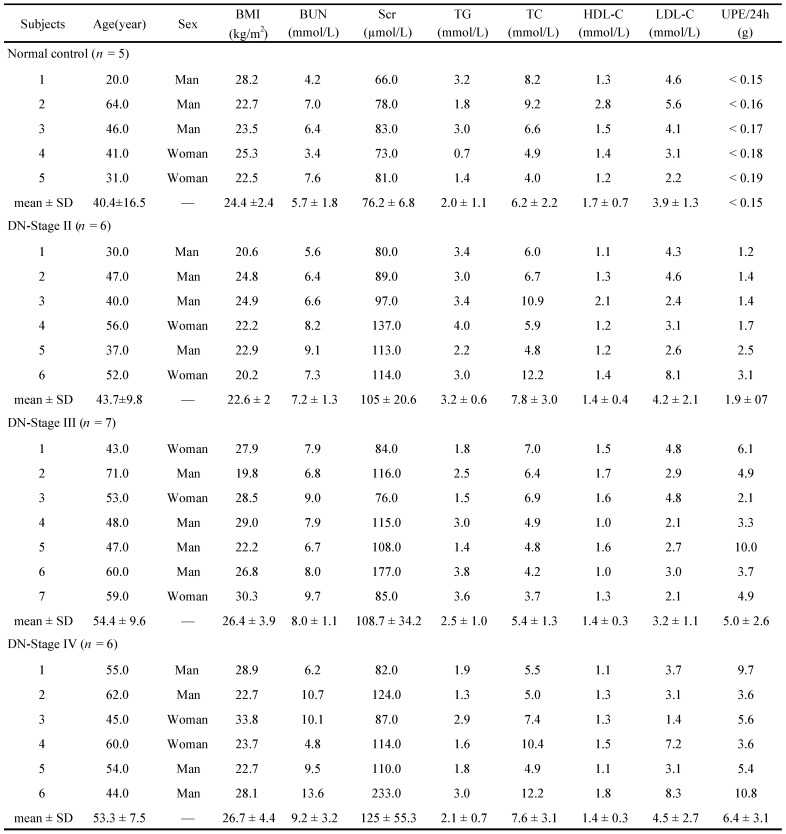
Baseline clinical data in healthy individuals and the subjects with DN of different pathological stages

BMI, Body Mass Index; BUN, blood urea nitrogen; Scr, serum creatinine; TG, triglycerides; TC, total cholesterol; HDL-C, high density lipoprotein cholesterol; LDL-C, low density lipoprotein cholesterol; UPE, urinary protein excretion; SD, standard deviation.

**Table 2:** Mouse gene primers for real-time PCR

Gene	Forward primer	Reverse primer
GPR43	ACCATCGTCATCATCGTTCA	ACGAAGCGCCAATAACAGAA
GPR41	CATGTGGTGGGCTATGTCAG	TGAGTCCAAGGCACACAAGT
Olfr78	CCACAATGCCCAAGATTCTC	AGAGCCACCATGCCTATTTG
IRS1	AGCTGCATAATCGGGCAAA	AGAGGCGGTAGATGCCAAT
IRβ	GGCCTGTCGCAACTTCTATC	GTATAGCCAGACGGGCACTC
Nephrin	GCCTCCTGACCATTGCTAA	TTTCCACTCCAGTCCTACCG
Collagen I	GATGGATTCCCGTTCGAGTA	TGCTGTAGGTGAAGCGACTG
α-SMA	CAGCAAACAGGAATACGACGAA	AACCACGAGTAACAAATCAAAGC
